# Eating disorders and diabetes: A meta-analysis

**DOI:** 10.1192/j.eurpsy.2021.963

**Published:** 2021-08-13

**Authors:** T. Mastellari, M. Speciani, F.F. Gelati, D. De Ronchi, F. Panariello, A.R. Atti

**Affiliations:** 1 Faculté De Médecine Henri Warembourg, University of Lille, Lille, France; 2 Department Of Biomedical And Neuromotor Sciences, University Of Bologna, Italy., University of Bologna, Bologna, Italy; 3 Mental Health, AUSL Bologna, Bologna, Italy

**Keywords:** eating disorders, disordered eating behaviours, diabetes

## Abstract

**Introduction:**

Diabetic patients are asked to focus on their eating habits and calories intake. Together with individual factors, this could increase the risk of developing Eating Disorders (ED) associated with diabetes. A score of 20 points at the Diabetes Eating Problem Survey-Revised (DEPS-R) scale is considered as a valid threshold to identify Disordered Eating Behaviours (DEB) in diabetic patients. DEB can be considered as altered eating behaviours not fully meeting criteria for ED. As DEB are not formally recognised as specific ED in DSM-5, there is a great risk of not detecting them, thus underestimate their consequences.

**Objectives:**

To meta-analyse literature on ED and DEB, when in comorbidity with Type 1 and Type 2 Diabetes Mellitus, focusing on pathological medical consequences.

**Methods:**

PRISMA guidelines were followed for this meta-analysis. Articles were identified in literature by searching into PubMed, PsycINFO and Embase.

**Results:**

1141 records were identified through database search. Figure 1 shows six studies comparing HbA1c % values for 2857 diabetic patients versus 752 diabetic patients with DEB. HbA1c % levels appear to be higher in patients with DEPS-R ≥ 20, compared to those with DESP-R scores below 20.

**Figure:**
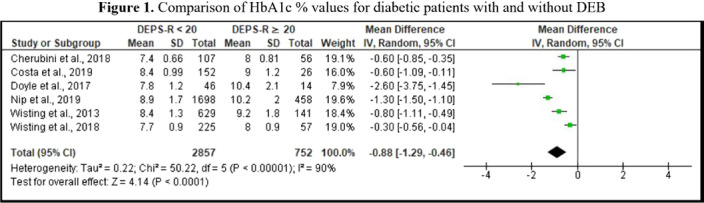

**Conclusions:**

Routine screening for DEB using DEPS-R scale could favour early identification of diabetic individuals, at risk for progression into a proper ED. Clinicians should be vigilant about potential DEB when patients show poor long-term glycaemic control; similarly, patients with a DEPS-R score over 20 points may require more frequent glycaemic checks. This could help prevent serious medical complications.

